# Catalytic Signature of a Heat-Stable, Chimeric Human Alkaline Phosphatase with Therapeutic Potential

**DOI:** 10.1371/journal.pone.0089374

**Published:** 2014-02-24

**Authors:** Tina Kiffer-Moreira, Campbell R. Sheen, Kellen Cristina da Silva Gasque, Mayte Bolean, Pietro Ciancaglini, Andrea van Elsas, Marc F. Hoylaerts, José Luis Millán

**Affiliations:** 1 Sanford Children's Health Research Center, Sanford-Burnham Medical Research Institute, La Jolla, California, United States of America; 2 Departamento de Química, FFCLRP-USP, Ribeirão Preto, São Paulo, Brazil; 3 AM-Pharma BV, Bunnik, The Netherlands; 4 Department of Cardiovascular Sciences, Center for Molecular and Vascular Biology, University of Leuven, Leuven, Belgium.; University of Dayton, United States of America

## Abstract

Recombinant alkaline phosphatases are becoming promising protein therapeutics to prevent skeletal mineralization defects, inflammatory bowel diseases, and treat acute kidney injury. By substituting the flexible crown domain of human intestinal alkaline phosphatase (IAP) with that of the human placental isozyme (PLAP) we generated a chimeric enzyme (ChimAP) that retains the structural folding of IAP, but displays greatly increased stability, active site Zn^2+^ binding, increased transphosphorylation, a higher turnover number and narrower substrate specificity, with comparable selectivity for bacterial lipopolysaccharide (LPS), than the parent IAP isozyme. ChimAP shows promise as a protein therapeutic for indications such as inflammatory bowel diseases, gut dysbioses and acute kidney injury.

## Introduction

Recombinant alkaline phosphatases (APs) (EC 3.1.3.1) show promise as therapeutic drugs for a variety of conditions ranging from soft-bone diseases such as hypophosphatasia (HPP) [1], inflammatory bowel diseases [2]–[4], gut dysbiosis [5], [6] and acute kidney injury [7]–[9]. Enzyme stability and substrate specificity are important parameters influencing the choice of isozyme to be used for a particular clinical indication. This paper describes the rationale for the construction of a chimeric enzyme (ChimAP) and its kinetic and biophysical properties.

APs are encoded by a multigene family [10] that in humans comprises four loci: *ALPL* encodes an enzyme expressed primarily in bone, liver and kidney, referred to as tissue-nonspecific AP (TNAP). The other three loci are highly homologous and are expressed in a tissue-specific manner. These genes, *ALPI, ALPP* and *ALPPL2*, encode intestinal AP (IAP), placental AP (PLAP) and placental-like or germ cell AP (GCAP), respectively. The known functions of these isozymes are quite different and relate to their substrate specificity [10]. Mutations in the human *ALPL* gene (*Alpl* in mice), cause hypophosphatasia (HPP), which is characterized by severe rickets, seizures and often perinatal death. The defects in HPP are caused by inadequate hydrolyses of the mineralization inhibitor [11] inorganic pyrophosphate (PP_i_) and pyridoxal-5′phosphate (PLP, a major isomer of vitamin B6), which are two physiological substrates of TNAP [10], [12]. In collaboration with Enobia Pharma (now Alexion Pharmaceuticals), our group developed the first therapeutic AP, a bone mineral-targeting recombinant TNAP (Asfotase alfa) that prevented seizures and all the skeletal and dental manifestations of HPP by normalizing PP_i_ and PLP metabolism [1], [13]–[16]. Clinical trials using asfotase alfa in patients with life-threatening HPP have shown remarkable results [17] (Clinicaltrials.gov Identifier NCT00744042) and are now continuing with adolescents and adults with HPP (ClinicalTrials.gov Identifiers: NCT00952484 and NCT01163149).

IAP has many interrelated functions in the gut. IAP participates in a rate-limiting step during fatty acid absorption with IAP knockout mice becoming obese [18] and developing metabolic syndrome under a high fat diet [19]. Importantly, IAP is a mucosal defense factor [5] maintained by enteral nutrition [20] that contributes to the establishment of the normal homeostatic gut microbiota [5], [6]. Furthermore, administration of bovine IAP prevents pathophysiological changes in mouse models of chronic colitis [21] by inhibiting pro-inflammatory pathways mediated by LPS, a gram negative bacterial endotoxin [20], and UDP [3] and preventing the development of metabolic syndrome in IAP knockout mice [22].

Here, we have developed a human recombinant AP that displays enhanced stability and good selectivity for LPS, the main pathophysiological substrate involved in acute kidney injury, inflammatory bowel diseases and gut dysbiosis. Our earlier studies on the structure of PLAP revealed an active site, with two active Zn^2+^ residues (Zn1 and Zn2) and a third metal ion site, occupied by Mg^2+^. While Zn2 is buried within the molecule, Zn1 is easily accessible and its reactivity can be modulated [23]–[25]. Both Zn1 and Zn2 coordinate with highly conserved amino acids [26] and our mutagenesis studies uncovered a major role for residue E429 in determining the affinity of Zn1, and thus the stability and catalytic properties of the PLAP active site as well as determining the overall thermal properties of the enzyme [25], [27]–[29]. We predicted that introducing the well-characterized crown domain of PLAP, harboring the critical E429 residue, into the IAP structure would improve enzyme stability while preserving or enhancing catalytic function of the resulting chimeric enzyme. Here, we show that ChimAP displays greatly increased heat stability, increased Zn^2+^ binding affinity, increased transphosphorylation, a higher turnover number and narrower substrate specificity, with comparable selectivity for bacterial LPS, than the parent IAP isozyme. Clinical trials using ChimAP have been initiated with the goal to treat patients with acute kidney injury (http://www.am-pharma.com/blog/2013/09/am-pharma-returns-to-clinic-with-phase-i-trial-of-new-recombinant-human-alkaline-phosphatase).

## Results

### 3D model of ChimAP

**Figure 2:**
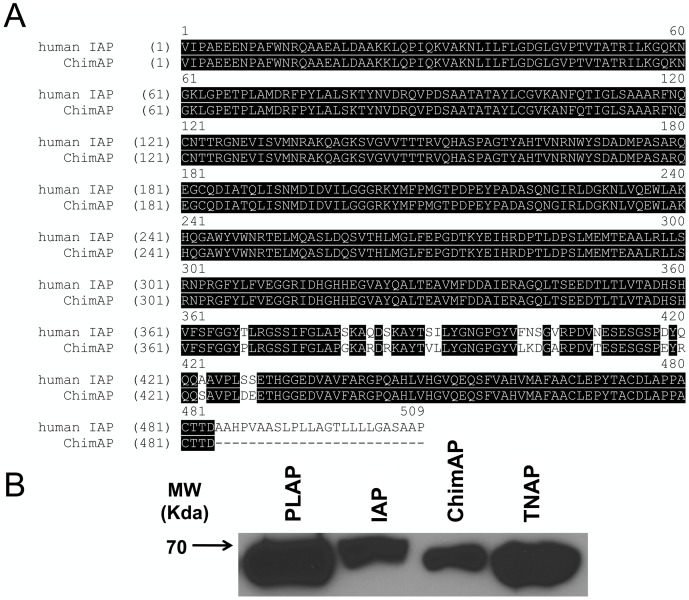
Human IAP and ChimAP sequence alignment and anti-FLAG western blot analysis of FLAG-tagged PLAP, IAP, ChimAP and TNAP. (**A**) Protein sequence alignment showing IAP and ChimAP sequence conservation. Substitutions are those introduced by replacement with the PLAP crown domain. (**B**) Western blot analysis showing that all four enzymes were secreted into the culture medium.

**Figure 1A** schematically illustrates where the PLAP amino acid segment was substituted into IAP (residues 360-430) to construct the chimeric enzyme ChimAP. The 3D positioning of the crown domain [23], [30] of ChimAP in the structural model of IAP illustrates how this compactly folded domain is a determinant of the IAP active site environment (**Figure 1B, C**). **Figure 2A** shows the complete sequence alignment of human IAP and ChimAP. Only 16 crown domain residues differ between them.

**Figure:**
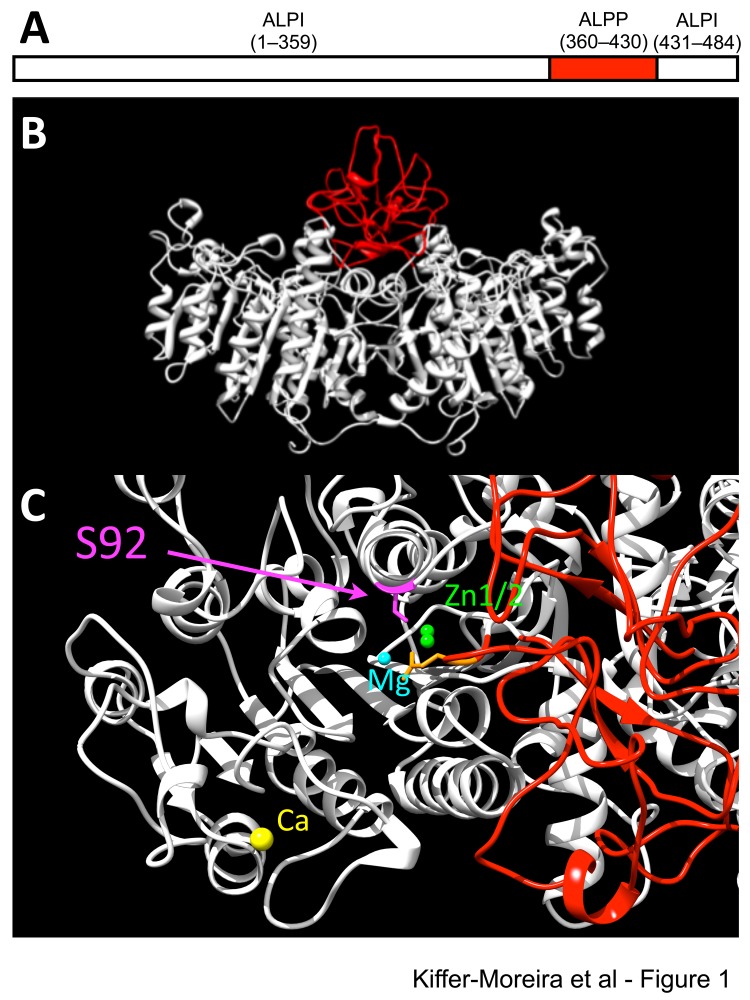
3D modeling of the structure of ChimAP. (**A**) Schematic representation of the fusion of the first 359 amino acids of the mature human IAP structure to residues 360–430 of the mature human PLAP sequence followed by residues 431–484 of the human mature IAP sequence. (**B**) 3D representation of the ChimAP structure based on homology to human PLAP, visualized and analyzed using Chimera v1.7 and Swiss-PdbViewer. (**C**) Top view of the ChimAP structure, indicating the active site serine. Active site metal ions are displayed. The crown domain is represented in red.

### Production of FLAG-tagged enzymes

To compare the kinetic properties of IAP, PLAP and ChimAP with TNAP, and to directly compare ChimAP with its parent enzymes, we added a FLAG-tag sequence to the IAP and ChimAP cDNAs, as we did previously to comparatively study PLAP and TNAP [26], [31]. The cDNAs were expressed in COS-1 cells and culture supernatant containing secreted enzymes was recovered. Successful expression and recovery were confirmed by anti-FLAG antibody western blot analysis (**Figure 2B**); all four monomers have comparable molecular masses of approximately 70 kDa.

### Active site stability

**Figure 3:**
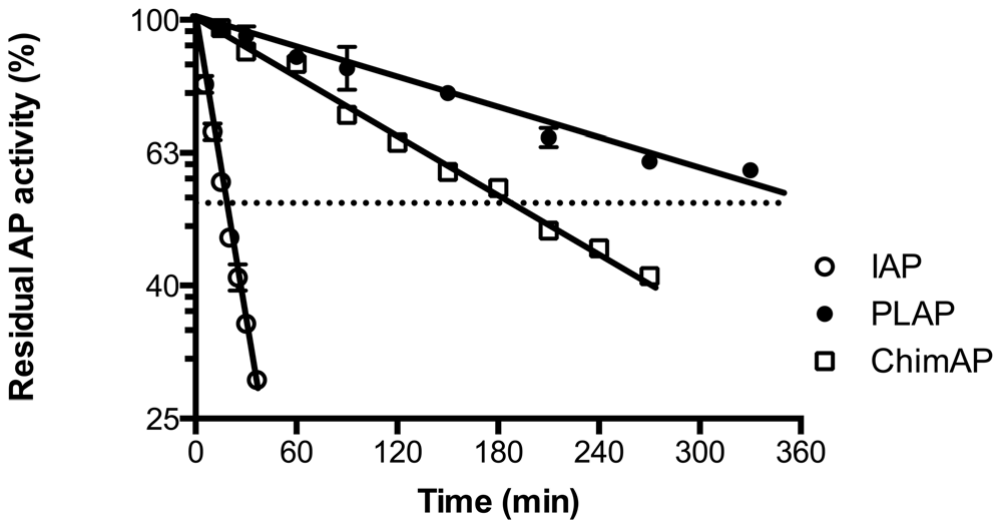
Time-course of AP activity disappearance upon dissociation of Zn^2+^ from the active site of IAP, PLAP and ChimAP at pH 7.4 in the presence of 250 µM EDTA. The slopes between these lines were highly significantly different (p<0.0001).

The flexible top loop, which is part of the crown domain, plays a major role in the AP structure-function relationship [23], [27]. To test our hypothesis that introducing the PLAP crown domain in to IAP would affect enzyme stability and kinetics of ChimAP, we first determined the rate of the catalytic Zn1 ion dissociation at physiological pH, in the presence of 250 µM EDTA, to preclude dissociated Zn^2+^ from rebinding. **Figure 3** shows that Zn1 ion dissociation [29] is a first order process, with half-lives of 20 min (IAP), 180 min (ChimAP) and 360 min (PLAP), corresponding to dissociation rate constants of 58×10^−5^ s^−1^, 6.4×10^−5^ s^−1^ and 3.2×10^−5^ s^−1^, respectively. This data shows that Zn1 readily dissociates from IAP, but is almost as tightly bound to its coordinating amino acids in ChimAP as in PLAP.

**Figure 4:**
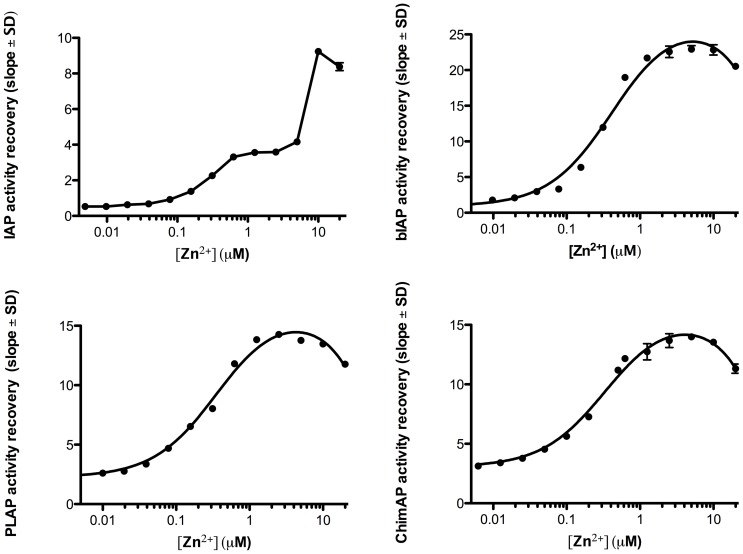
Dose-dependent reconstitution of AP activity upon addition of Zn^2+^ to Chelex-treated alkaline phosphatases at pH 7.4.

Directly adding the active site metal chelator agent Chelex to all three enzymes removed Zn^2+^ with similar inactivation kinetics (not shown). During this time frame, enzymes were quickly regenerated upon adding Zn^2+^, which dose-dependently reconstituted the active site in IAP, PLAP, the highly active bovine IAP (bIAP) II [32], and ChimAP, reaching steady-state enzyme activity in a few minutes (**Figure 4**). Zn^2+^ reconstitution occurred between 0.1–1 µM Zn^2+^, with the exception of IAP where an additional sharp rise of enzyme activity occurred between 5–20 µM Zn^2+^. These findings suggest similar association rates constants in all cases, i.e. full active site access, even though the profile for IAP suggests additional conformational changes occurring in demetalated IAP [33]. To minimize protein monomerization and incomplete recovery during IAP remetalation, reconstitution was initiated before full enzyme inactivation was achieved, explaining the residual activity at 0.01 µM Zn^2+^ in **Figure 4**. Absence of Mg^2+^ can explain the drop in enzyme activity at [Zn^2+^]>10 µM, because Zn^2+^ binding to the Mg^2+^ site inhibits PLAP activity [34].

### Kinetics parameters with standard pNPP substrate at pH 9.8 and 7.4

**Figure 5:**
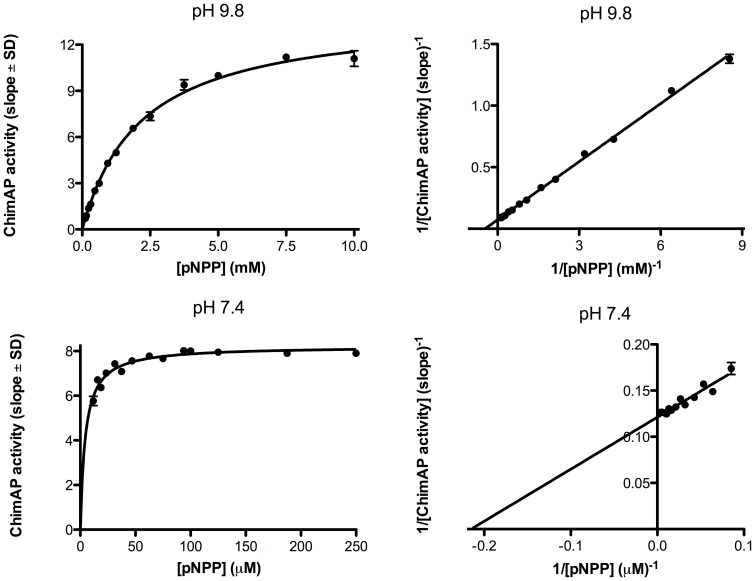
Differences in catalytic behavior for ChimAP at alkaline and physiological pH, analyzed via pNPP substrate saturation curves and corresponding Lineweaver-Burk plots.

The baseline kinetic characterization of ChimAP at alkaline pH in a transphosphorylating buffer is shown in **Figure 5**, with pNPP as a substrate. This figure illustrates that the determination of catalytic AP properties at alkaline pH bears little relevance to their application at physiological pH. **Table 1** summarizes the kinetic constants for the substrate pNPP at both pHs for all enzymes. At pH 9.8 IAP, TNAP and ChimAP have comparable catalytic efficiency. However, closer analysis shows that K_m_ and k_cat_ are about five-fold higher for ChimAP than for IAP. Interestingly, despite its low K_m_ at pH 7.4, ChimAP has k_cat_ values that are higher than IAP, and almost as high as the highly active bIAP II (123 s^−1^). Overall, the catalytic efficiency of ChimAP was the highest at pH 7.4 (**Table 1**), justifying further analysis of physiological substrates at this pH, using pNPP as a reference.

**Table 1:** Enzyme constants (± SD) using the artificial substrate p-nitrophenylphosphate (pNPP) at alkaline and physiological pH

	Enzyme	K_m_ (µM)	k_cat_ (s^−1^)	k_cat_/K_m_ (µM^−1^ s^−^)
**pH 9.8**	IAP	260±30	753±216	2.9
	PLAP	430±50	460*	1.07
	ChimAP	1,410±260	3,282±651	2.3
	TNAP	370±60	1,102*	3.0
**pH 7.4**	IAP	24±6.5	22.5±0.66	0.93
	PLAP	18.7±2.2	7.7±0.3	0.41
	ChimAP	4.11±0.27	46±1.4	11.2
	TNAP	3.7±0.9	7.5±0.22	2.0

*Historical values, used as internal standards, were taken from our previous study[50]. k_cat_ ∼123 s^−1^ and ∼5,000 s^−1^ for highly active bIAP II at pH 7.4 and pH 9.8, respectively[32]. k_cat_ values were determined from the rate of p-NPP formation at pH 9.8 and from the rate of P_i_ formation at pH 7.4.

### Kinetics parameters with physiological substrates

**Figure 6:**
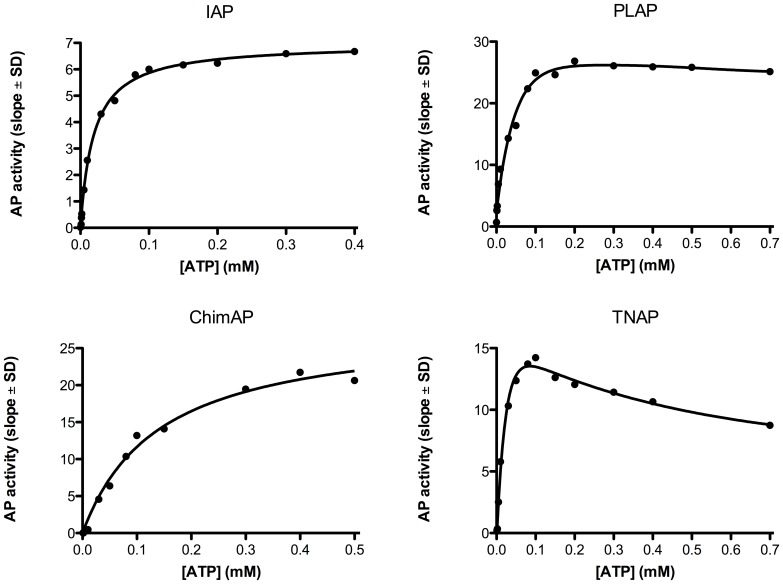
Efficiency of IAP, PLAP, ChimAP and TNAP at pH Plots are representative of three separate experiments.

The phosphohydrolase properties of ChimAP were investigated for physiological substrates implicated in bone metabolism, inflammation and seizures, specifically the mineralization inhibitor PP_i_, the nucleotides ATP, ADP and AMP, LPS and the vitamin B_6_ vitamer PLP. **Figures 6** **–** **9** show relative binding saturation for all enzymes, using ATP, PP_i_, LPS and PLP as substrates, which illustrates that LPS and PLP are good substrates for ChimAP. These findings are summarized in **Table 2**, including data for ADP and AMP. The data reveal comparable to superior catalytic efficiencies for IAP and TNAP, consistent with the role of the latter in bone metabolism, where it is a potent pyrophosphatase [35]. Overall, the k_cat_ constants were comparable to those measured for pNPP at pH 7.4 following the same methodology. Small differences were found for all three nucleotides, but most striking was that the K_m_ of ChimAP is especially high with PP_i_, and lower with ATP as a substrate. At the same time, k_cat_ for PP_i_ is low, categorizing ChimAP, together with PLAP, as an enzyme with a poor catalytic efficiency for PP_i_ hydrolysis. Importantly, at physiological pH with all other substrates, ChimAP displayed equal or greater k_cat_ constants than IAP (**Table 2**).

**Figure 7:**
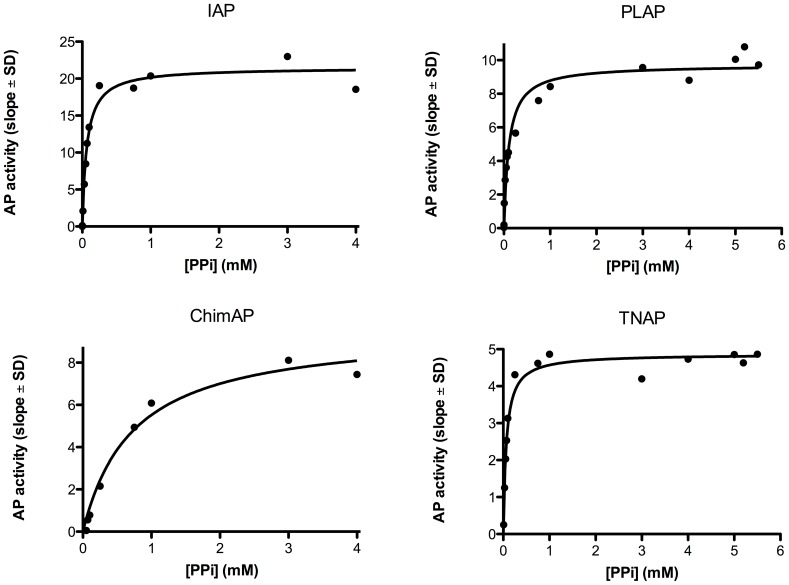
Efficiency of IAP, PLAP, ChimAP and TNAP using the physiological substrate PP_i_, measured as the rate of phosphate formation versus substrate concentration. Plots are representative of three different experiments.

**Figure 8:**
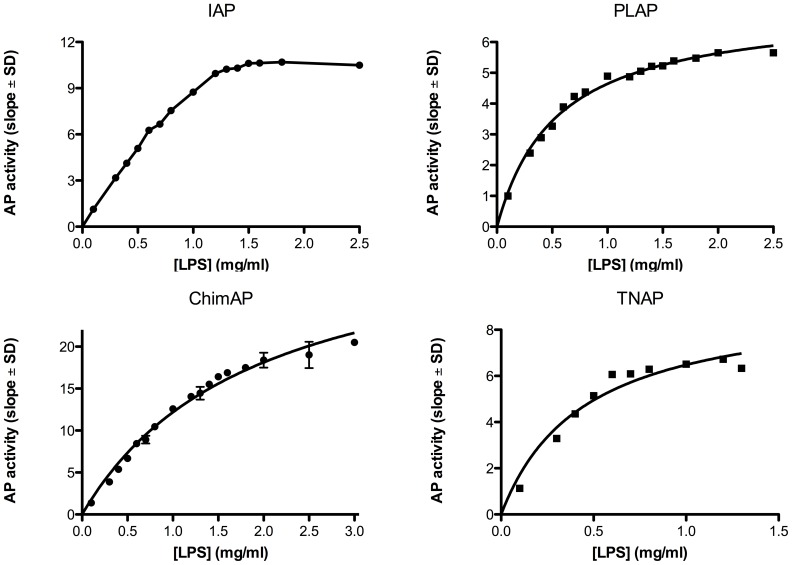
Efficiency of IAP, PLAP, ChimAP and TNAP using the pathophysiological substrate LPS, measured as the rate of phosphate formation versus substrate concentration. Plots are representative of three different experiments.

**Figure 9:**
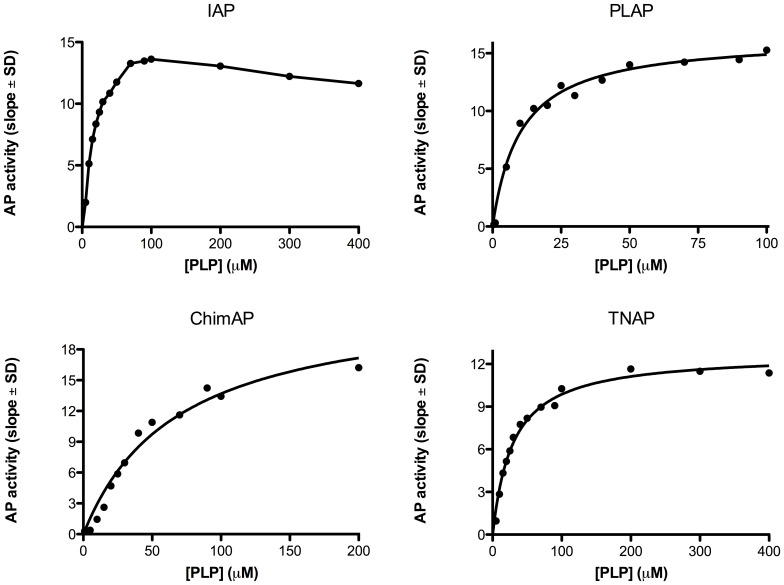
Efficiency of IAP, PLAP, ChimAP and TNAP using the physiological substrate PLP, measured as the rate of phosphate formation versus substrate concentration. Plots are representative of three different experiments.

**Table 2:** Kinetic parameters (± SD) of recombinant enzymes with physiological substrates at physiological pH.

Enzyme	K_m_ (µM)	k_cat_ (s^−1^)^1^	k_cat_/K_m_ (µM^−1^ s^−1^)
Substrate: ATP			
IAP	31±22	18.9±0.4	0.61
PLAP	32±7	1.9±0.18	0.06
ChimAP	113±16	21±2.1	0.19
TNAP	15±8.7	8.9±0.27	0.6
Substrate: ADP			
IAP	20±5.7	22.6±0.99	1.13
PLAP	6.8±3	6.3±0.27	0.93
ChimAP	44±6	36±1.0	0.82
TNAP	3.9±1.3	5.6±0.08	1.4
Substrate: AMP			
IAP	43±7	22.6±0.09	0.52
PLAP	9±2.5	7.9±0.1	0.88
ChimAP	71±17	35±0.06	0.49
TNAP	7.7±1.4	7.1±0.33	0.92
Substrate: PP_i_			
IAP	66±0.2	36.6/2±4.9/2^2^	0.55/2
PLAP	149±59	1.70/2±0.01//2^2^	0.011/2
ChimAP	773±55	15.5/2±1.6/2^2^	0.02/2
TNAP	69±3.8	22.0/2±0.4/2^2^	0.32/2
Substrate: PLP			
IAP	31±22	17.8±0.39	0.57
PLAP	9.5±1	7.52±0.28	0.79
ChimAP	40±24	32.4±0.75	0.81
TNAP	17±10	5.8±0.02	0.34
Substrate: LPS	(mg/ml)	s^−1^	(s^−1^ mg/ml^−1^)
IAP	0.62±0.02	19.1±2.3	31
PLAP	0.48±0.02	2.3±0.09	4.8
ChimAP	0.79±0.2	16.4±0.83	21
TNAP	0.35±0.02	7.5±0.73	21

1 k_cat_ values were determined from the rate of P_i_ formation.

2 As the detection of two P_i_ corresponds to one cleavage event, the actual rate is only half of the calculated number of P_i_ molecules released.

### Enzyme inhibition

**Figure 10:**
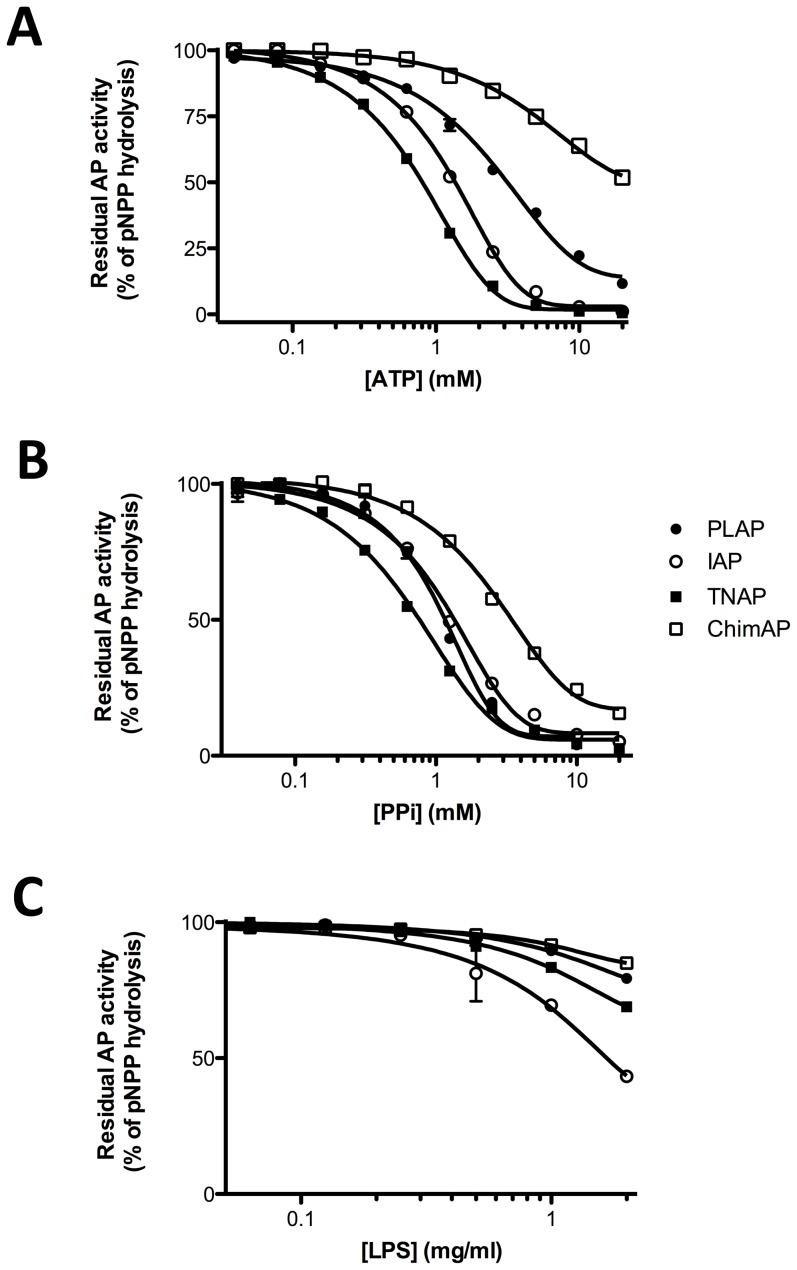
Integrated competition experiments for the enzymes specified between 0.1_i_ and LPS at the indicated concentrations.

**Figure 11:**
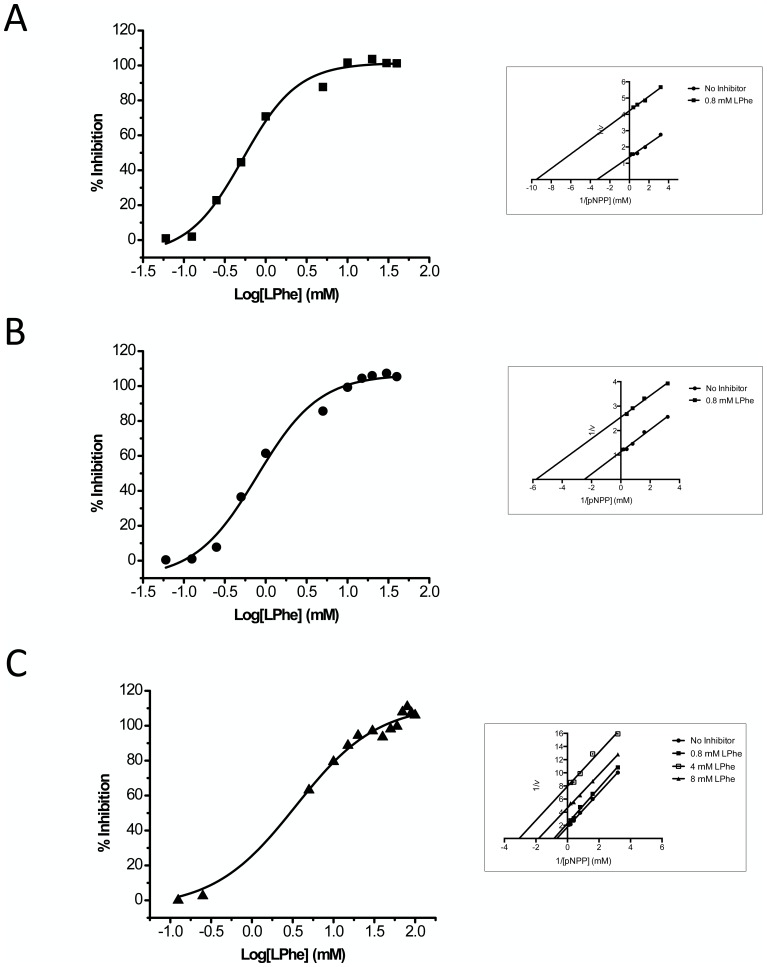
Active site inhibition of FLAG-tagged IAP (A), PLAP (B) and ChimAP (C) activity at pH 9.8 versus pNPP concentration for the indicated concentrations of the uncompetitive inhibitor L-Phe. The lines represent fitted inhibition curves. Inset: respective double-reciprocal plots of FLAG-tagged PLAP, IAP and ChimAP activity in the presence or absence of the indicated concentrations of L-Phe.

Integrated inhibition experiments were carried out, in which ATP, PP_i_ and LPS were allowed to compete with a fixed concentration of pNPP (0.1 mM). **Figure 10** illustrates that both ATP and PP_i_ most easily displace pNPP from TNAP and LPS displaces pNPP most easily from IAP, at pH 7.4. Although TNAP and ChimAP have a comparable K_m_ for pNPP, ATP and PP_i_ displace pNPP from ChimAP less efficiently, consistent with the highest K_m_s of ChimAP for these substrates. Likewise, LPS displaces pNPP from ChimAP with the lowest efficiency. Active site catalysis was also probed using the established uncompetitive active site inhibitors L-Phe and L-hArg (**Figure 11**). **Table 3** shows that the inhibition constant for L-Phe is about six-fold higher for ChimAP than for IAP. Also, ChimAP was less inhibited by L-hArg than PLAP and IAP.

**Table 3:** Uncompetitive inhibition with L-amino acids, expressed at IC_50_ (± SD).

Enzyme	IC_50_ L-Phe (mM)	IC_50_ L-hArg (mM)
IAP	0.53±0.07	40.2±0.2
PLAP	0.76±0.08	61.7±3.3
ChimAP	3.40±0.11*	>100*

*p<0.005 vs corresponding IC_50_ for IAP and PLAP.

### Enzyme stability

**Figure 12:**
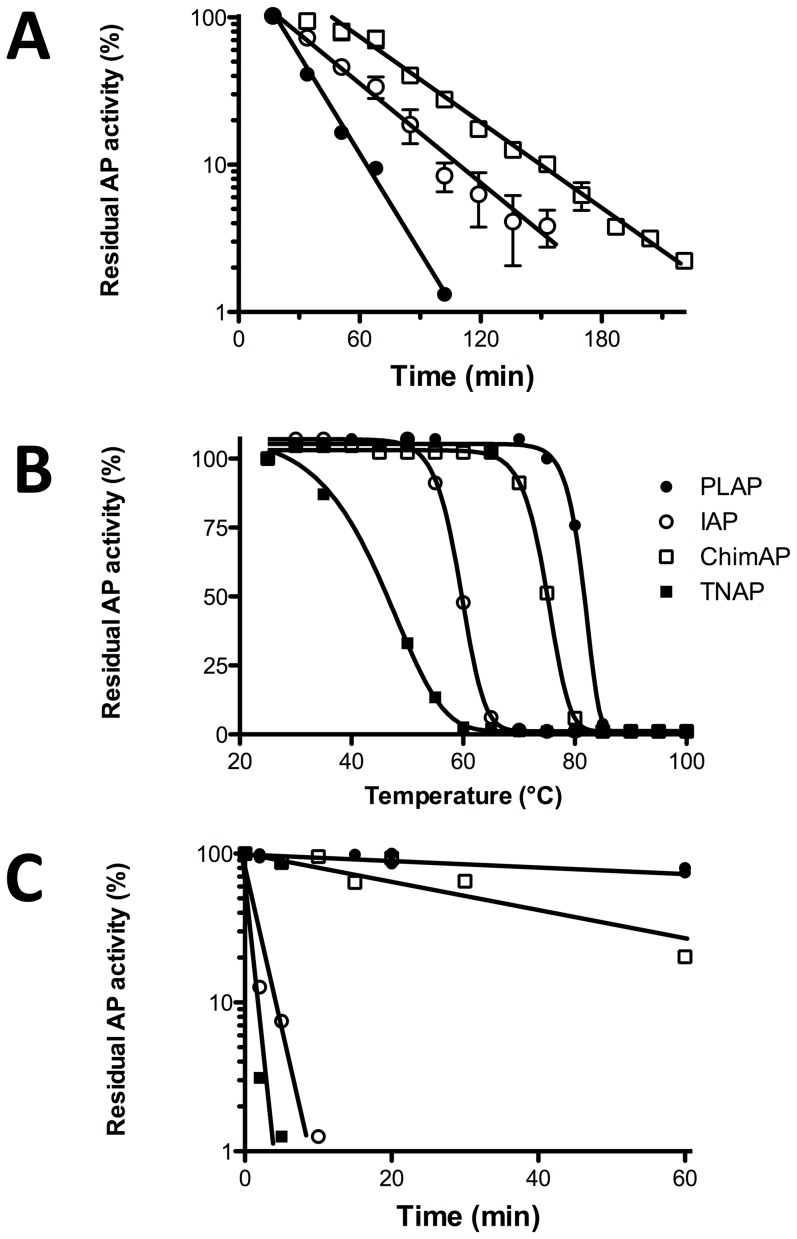
Enzyme stability studies. (**A**) Plot of residual enzyme activity in the exponential phase of enzyme inactivation for FLAG-tagged PLAP, IAP, ChimAP and TNAP, showing the effect of the denaturing agent guanidinium hydrochloride. (**B, C**). Lines in B and C are representative of three separate experiments. Heat inactivation of FLAG-tagged enzymes PLAP, IAP, ChimAP and TNAP. The enzymes were incubated for 10 min at different temperatures (25–100°C) (**B**) or for different time intervals at 65°C (**C**).

The impact of the crown domain exchange on the overall stability of the enzyme was investigated via unfolding in guanidinium hydrochloride and by heat inactivation studies. **Figure 12A** shows ChimAP denaturation as a function of time, when incubated in 3.8 M GndCl, first slowly but then exponentially. The slopes of the denaturation rates were highly statistically different (p<0.0001), but the value for the fractional disappearance rate per minute of ChimAP activity (9.5±0.24×10^−3^) was considerably closer to the inactivation rate of IAP (12.2±0.97×10^−3^) than to that of PLAP (21.5±0.59×10^−3^) (**Table 4**). Consistent with the 95% sequence homology between IAP and ChimAP, these findings confirm that the overall structure of ChimAP resembles that of IAP. PLAP is extremely resistant to heat inactivation at high temperatures (**Figure 12B**) and showed the highest resistance to heat of the APs studied (**Table 4**). In contrast, IAP and TNAP are relatively heat labile. ChimAP displays strong heat stability, almost comparable to that of PLAP. Consistent with the existence of a stable Zn environment in the active site of ChimAP (**Figure 3**), these findings indicate that the crown domain enhances stability to the active site region of ChimAP. Detailed inactivation kinetics at 65°C confirmed that IAP and TNAP were inactivated rapidly, following first order kinetics, whereas the slow loss of ChimAP activity matched the extreme heat stability of PLAP (**Figure 12C**, **Table 4**).

**Table 4:** Enzyme stability when exposed to guanidinium chloride (GndCl) or heat.

Enzyme	*Half-life 3.8 M GndCl (min)	Half-life 65°C (min)	50% Inactivation (°C)
IAP	8.4	3.2	63.8
PLAP	6	>60	83.4
ChimAP	9	35	78.8
TNAP	ND	<1.0	48

*Calculated from the exponential decay phase in **Figure 4A** of main paper. ND: not determined. Computed inactivation rates for the exponential denaturation rates for IAP and ChimAP were similar (see text).

## Discussion

**Figure 13:**
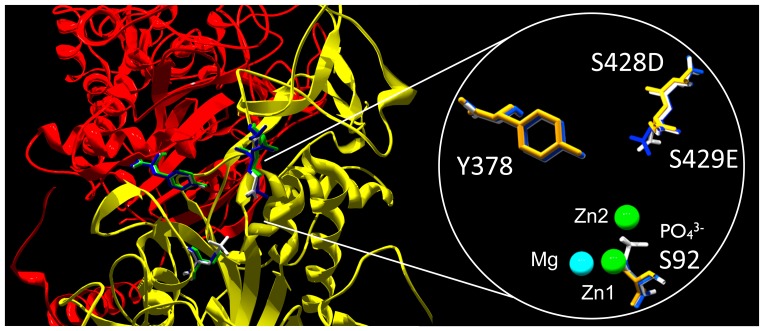
Modeling of the predicted effect of the S429E substitution on the active site environment of the superimposed structures of IAP, PLAP and ChimAP. IAP, PLAP and ChimAP are shown in orange, white and blue, respectively.

Previous work in our laboratory has demonstrated the crucial importance of the flexible surface loop of mammalian APs in determining isozyme-specific properties in this enzyme family [27]. Replacement of the flexible surface loop of TNAP with that of PLAP conferred heat stability properties and stabilized uncompetitive inhibitors in the active site of TNAP [26], [27], [29], [31]_ENREF_26_ENREF_27. Furthermore, the stability of the catalytic metal ions in PLAP was lost upon introduction of a E429G substitution in that loop [29]. In the current paper we have constructed the chimeric ChimAP recombinant enzyme with improved stability and kinetic properties towards physiological substrates. Replacing the entire crown domain of human IAP with the equivalent residues of PLAP (16 amino acid substitutions) conferred greatly enhanced heat and Zn stability to ChimAP, approaching that of PLAP, while also maintaining and enhancing the substrate specificity of ChimAP for the physiologically relevant substrate LPS. The substituted crown domain markedly influenced the active site localization of Zn^2+^, in turn enhancing enzyme activity. In addition to the amino acid residues coordinating Zn^2+^ [26], the stability of the Zn^2+^ ions is lost by mutation of E429G in PLAP [29]. Consistent with those findings, the increase in Zn^2+^ stability observed between IAP and ChimAP is explained by the presence of E429 in ChimAP (**Figure 13**).

The presence of E429 is also the possible explanation for the kinetic behavior of ChimAP with pNPP at pH 9.8 and with physiological substrates at physiological pH. Enzyme catalysis by AP can be represented schematically as follows:
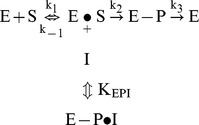
where E is enzyme, S is substrate and P is product, with k_cat_ = k_2_/(1+k_2_/k_3_) and K_m_ = (k_−1_+k_2_)/k_1_(1+k_2_/k_3_). Furthermore, the catalytic efficiency corresponds to k_cat_/K_m_ = k_1_/(1+k_−1_/k_2_) and the inhibition constant for uncompetitive inhibition K_i_ = K_EPI_(1+k_3_/k_2_).

One concern that we had was that the PLAP crown domain residue E429 would cause active site hindrance in ChimAP, and be the cause of the high K_m_ for pNPP measured at pH 9.8. Yet, the k_cat_ was similarly increased in these conditions, hence resulting in a comparable k_cat_/K_m_ ratio as for IAP. Since both K_m_ and k_cat_/K_m_ depend on the active site entrance constant k_1_, steric hindrance can be excluded, because it would have affected both parameters. Since in the overall reaction k_−1_<<k_2_, this conclusion suggested the rate of phosphorylation of the active site serine residue (k_2_) or the rate of transphosphorylation onto DEA (k_3_) to be affected in ChimAP. A rise in k_2_ can be excluded, because it would also lead to a lower K_i_ (at constant K_EPI_ in the absence of active site hindrance). Therefore, our findings are compatible with an increase in k_3_, explaining a similar rise in k_cat_ and K_m_ and a parallel rise of K_i_. In other words, the increased Zn stability results in more efficient intramolecular hydrolysis of the phospho-enzyme complex and transfer of the hydrolyzed P_i_ group to the transphosphorylation buffer DEA. In contrast, at pH 7.4, transphosphorylation is virtually absent and the same relationship no longer applies. Moreover, with various physiological substrates, no single trend was observed, i.e. k_cat_ and K_m_ did not consistently change in the same direction.

At pH 7.4, ChimAP is characterized by three unique properties: a) K_m_ values are higher than for IAP with all substrates, b) k_cat_ values are also slightly higher, except with PP_i_ as a substrate, and c) ChimAP shows selectivity for some substrates. Overall, catalytic efficiencies were comparable for ChimAP and IAP, but in some cases were lower than for TNAP. PP_i_ appeared to be a poor substrate (high K_m_ and low k_cat_) for ChimAP resulting in a low catalytic efficiency. It is clear from the rise in K_m_ and the drop in k_cat_ that, in addition to an effect on k_2_, k_1_ is also lowered in ChimAP when PP_i_ is the substrate, as compared to IAP. It is noteworthy that PLAP is a poor enzyme with all the the presently studied physiological substrates; it had the lowest catalytic rate constants for all substrates. The dual negative PLAP-like amino acid replacements S428D and S429E in ChimAP may explain this finding, as PLAP also hydrolyses the highly negatively charged PP_i_ poorly. We surmise that the S429E substitution in the vicinity of the ChimAP active site reduces active site entrance of strongly negatively charged substrates such as ATP, and even more so for the smaller but more densely negative PP_i_. However, at physiologically low substrate concentrations (i.e. below the respective K_m_), ChimAP can hydrolyze some substrates at least as effectively as IAP.

Our studies reveal a relative preference of IAP and ChimAP for the physiological substrates LPS>PLP>ATP>PP_i_, while the reverse is true for TNAP, with a preference order of PP_i_>PLP = ATP>LPS. This substrate preference correlates well with the known biological function of the native isozymes, where IAP is involved in maintaining gut mucosal barrier function via LPS detoxification. Here, we have not attempted to compare different types of LPS, as the main objective has been to engineer the enzyme to improve its biophysical properties and narrow down specificity. However, we previously reported that bIAP [3], murine TNAP and IAP [36] are able to dephosphorylate *E.coli* LPS O55:B5. Also, human PLAP is able to dephosphorylate LPS (Re595) from *S. minnesota* as well as *E. coli* LPS O55:B5 [37]. Thus, it is likely that ChimAP will be active towards LPS produced by a wide number of bacteria. Neither have we systematically compared LPS and monophosphoryl-lipid A (MPLA) as substrates for ChimAP. However, Bentala et al [37] demonstrated that LPS, but not MPLA, from *S. minnesota* Re595, is a good substrate for human PLAP. Given that ChimAP has acquired many biophysical characteristics of PLAP, it is likely that the discrimination between di- and monophosphorylated lipid A will also be displayed by ChimAP.

TNAP is required during the physiological mineralization of skeletal and dental tissues via its ability to control the P_i_/PP_i_ ratio with its pyrophosphatase function [38], [39]. TNAP is also required in the metabolism of PLP, the most important vitamin B_6_ vitamer, which is an essential cofactor for neurotransmitter synthesis [40], [41] and also in establishing an appropriate ATP/adenosine ratio for nociception in the dorsal root ganglia [42] and in neonatal blood [43]. PLAP appears relatively inefficient in hydrolyzing most of these physiological substrates, and it is still unclear what its biological role is. However, the fact that large amounts of PLAP are expressed in the human placenta from the second trimester of pregnancy until term may compensate for the suboptimal kinetic properties of this isozyme [10]. In contrast, the highly specific presence of IAP in the brush border of the S3 segment of the human renal proximal tubule [44] has been an enigma for over two decades. In view of the high IAP catalytic efficiency shown in the present study, it seems logical that the highly vulnerable S3 segment of the proximal tubule expresses an enzyme that hydrolyses phosphorylated compounds, such as LPS and ATP, very efficiently to avoid cellular damage upon re-absorption of such substrates. The kinetic observations in this study parallel what we know about the evolutionary relationship between these isozymes, with *ALPL* (encoding human TNAP) being the most ancestral of the AP genes, followed by a clustering of all the intestinal isozymes in all species studied (*ALPI* encoding human IAP) with functions clearly unrelated to those of TNAP. The latest evolutionary members of this family of isozymes, PLAP (*ALPP*) and the closely related GCAP (*ALPPL2*), have unknown biological functions and show a marked degree of genetic variability, consistent with their late appearance during evolution [10].

In conclusion, ChimAP is an enzyme with increased stability, increased Zn^2+^ binding affinity, increased transphosphorylation, a higher turnover number and narrower substrate specificity with selectivity for bacterial LPS, compared to the parent IAP isozyme. In fact, ChimAP displays turnover rates for various pathophysiologically relevant substrates with values that compare well with the rates displayed by the AP isozyme with the highest turnover numbers known to-date, that of the *Bos taurus* bIAP II isozyme [32]. The increased stability of ChimAP should facilitate the development of formulations suitable not only for intravenous administration, as may be required for the treatment of acute kidney injury, but also for oral administration suitable for indications such as inflammatory bowel disease and a variety of gut dysbioses. ChimAP has recently been shown to be efficacious in two rat models of acute kidney injury [45]. A single iv dose of ChimAP (1000 U/Kg) was administered to Wistar rats within 30 minutes after ischemia- reperfusion (I-R) or LPS injection to induce AKI. ChimAP demonstrated pharmacological effect within the 3 hours of the experiment that included suppression of acute inflammation in the afflicted kidney and inhibition of tissue injury [45]. Clinical trials using ChimAP have been initiated with the goal to treat patients with acute kidney injury (http://www.am-pharma.com/blog/2013/09/am-pharma-returns-to-clinic-with-phase-i-trial-of-new-recombinant-human-alkaline-phosphatase).

## Methods

### Protein structure modeling

The primary sequences of human IAP and ChimAP were submitted to the SWISS-MODEL server [46] to model their tertiary structures, based on homology to human placental alkaline phosphatase (1ZED). The resulting molecular structures were visualized and analyzed using Chimera v1.7 [47] and Swiss-PdbViewer [48].

### Protein expression

Expression plasmids containing secreted, FLAG epitope-tagged PLAP, IAP and TNAP were described previously [26], [31]. ChimAP was constructed by substituting residues 360–430 of the mature IAP structure for the corresponding residues of mature PLAP. All four FLAG-tagged enzymes were transiently transfected into COS-1 cells by electroporation, then grown in DMEM medium for 24 h, as previously described [49], when the medium was replaced with serum-free Opti-MEM (Life Technologies, Grand Island, NY). Opti-MEM containing secreted proteins was collected 60 hours after transfection, then filtered through a 2-µm cellulose acetate filter and dialyzed against TBS containing 1 mM MgCl_2_ and 20 µM ZnCl_2_.

### Western blot analysis

FLAG-tagged enzyme samples were denatured in 2% SDS and 0.025% β-mercaptoethanol and loaded on 8–16% acrylamide Tris-glycine gels (Invitrogen, Carlsbad, CA). Gels were transferred to Optitran nitrocellulose membranes [49] (Schleicher & Schuell Bioscience, Keene, NH), and blotted membranes were stained with Ponceau S to ensure efficiency of transfer. Washed membranes were blocked with SuperBlock reagent (Pierce Biotechnology, Rockford, IL) prior to incubation with mouse monoclonal anti-FLAG primary antibody (Sigma-Aldrich, St. Louis, MO), then peroxidase-labeled goat anti-mouse IgG (Calbiochem - Merck4Biosciences) as a secondary antibody.

### Enzyme kinetics

To measure the relative catalytic activities of FLAG-tagged enzymes using different substrates, microtiter plates were coated with anti-FLAG M2 antibody (Sigma-Aldrich, St. Louis, MO) at 0.2–0.6 µg mL^−1^. These plates were incubated with saturating concentrations of FLAG-tagged PLAP, IAP or ChimAP for 3 h at room temperature, after which plates were washed with PBS, containing 0.008% Tween-80 and the relative activities for various substrates (p-nitrophenylphosphate (pNPP) and physiological substrates, see below) were compared for the M2-saturated enzymes. The activity of bound enzymes was measured as the absorbance at 405 nm (*A*_405_) as a function of time at 25°C, using pNPP (0.02–20 mM) as a substrate, at pH 9.8 in 1 M diethanolamine (DEA) buffer containing 1 mM MgCl_2_ and 20 µM ZnCl_2_, or at pH 7.4 in 50 mM Tris-HCl buffer, 100 mM NaCl, containing 1 mM MgCl_2_ and 20 µM ZnCl_2_, as indicated. Recordings for kinetic analysis were selected from those parts of the curve where *A*_405_ versus time was linear; initial rates were calculated over a period of up to 1 h, excluding the first 5 min. 1/*v* versus 1/[substrate] Lineweaver-Burk plots were constructed and linear regression was performed, calculating slopes (± SD) and intercepts (± SD), in Prism v3.0a (GraphPad Software) to determine K_m_ values. Catalytic rate constants (k_cat_) were derived by comparison with the values measured for PLAP-FLAG, which was used as an internal standard with a known k_cat_ for pNPP (460 s^−1^) at pH 9.8[50], at equal amounts of M2-saturated FLAG-enzymes.

Similarly, known concentrations of commercial recombinant human IAP (Sino Biological Inc., Beijing, China), human PLAP (Sigma-Aldrich, St. Louis, MO) and recombinant bovine intestinal alkaline phosphatase (Roche Diagnostics, Indianapolis, IN) were incubated with pNPP (0.1–10 mM) at pH 9.8, and Lineweaver-Burk plots were constructed, from which K_m_ was determined. Numerical values for k_cat_ at pH 9.8 were calculated by dividing the rate measured for 20 mM pNPP in 1 M diethanolamine buffer containing 1 mM MgCl_2_ and 20 µM ZnCl_2_ by the known enzyme concentration, using a molar extinction coefficient ε = 18,000 M^−1^ cm^−1^ for p-nitrophenol, at pH 9.8.

For the direct measurements of k_cat_ at physiological pH, we used lipopolysaccharides (LPS) from *Escherichia coli* O111:B4 (Product number L 2630, Sigma, St. Louis, USA). This LPS preparation is in aggregated form and was dissolved as recommended in the manufacturer's data sheet. All other substrates, PP_i_, pyridoxal-5′-phosphate (PLP), ATP, ADP and AMP were also from Sigma-Aldrich (St. Louis, MO). Hydrolysis of the physiological substrates was measured at pH 7.4 in standard assay buffer (50 mM Tris-HCl buffer, 100 mM NaCl, 1 mM MgCl_2_ and 20 µM ZnCl_2_). The concentration of released phosphate was determined using P_i_ ColorLock Gold (Innova Biosciences) by measuring absorbance at 650 nm (*A*_650_). Standard curves constructed for increasing concentrations of phosphate were linear between 0–50 µM and all experiments were designed to fall within this range of hydrolyzed phosphate concentrations. Molar reaction rates, expressed as [P_i_] s^−1^ were calculated for the indicated substrate concentration range and were fitted to a one-binding site model (GraphPad Prism) versus [substrate] to calculate K_m_ (Lineweaver-Burk plots were not applied, because of lack of precision of reciprocal conversions at very low substrate concentrations). To compute k_cat_, the calculated rate [P_i_] s^−1^ was divided by the known enzyme concentration. Because of the lower molar extinction coefficient for pNPP at pH 7.4, the hydrolysis of pNPP at pH 7.4 was analyzed using the P_i_ ColorLock Gold method.

The following substrate concentrations were used: [pNPP] = 250 µM, [ATP] = 500 µM, [ADP] = 500 µM, [AMP] = 500 µM, [PP_i_] = 5 mM for ChimAP and 2.5 mM for the other enzymes, [PLP] = 400 µM and [LPS] = 2.5 mg mL^−1^ for ChimAP and 1.25 mg mL^−1^ for the other enzymes. The soluble enzyme concentration was about 1 nM and incubation times ranged from 15–30 min, depending on the catalytic efficiency for each enzyme. To ensure steady-state conditions and to correct for non-specific substrate signals in the P_i_ ColorLock Gold method, an early reading (at 5 min) was subtracted from the later reading, and Δ*A*_650_ was measured on the corresponding P_i_ standard curve, constructed for each experiment separately. All experiments were carried out three to five times and the derived constants are reported as mean ± SD.

To measure the heat stability, enzymes were incubated at 65°C in 1 M DEA (pH 9.8) containing 1 mM MgCl_2_ and 20 µM ZnCl_2_. Samples were removed at different time points and placed on ice, then residual activity was measured as described above with 10 mM pNPP. Enzymes were also incubated in this buffer for 10 min at increasing temperatures (25–100°C) and residual activity was measured in the same manner. For experiments where the stability of enzymes was measured in the presence of a denaturing agent, assays were performed in standard assay buffer containing 3.8 M guanidine hydrochloride. The absorbance was recorded continuously in the presence of 20 mM pNPP for up to 4 h and slopes were calculated at different time points. Slopes were plotted semi-logarithmically versus time, to determine the half-life (t_1/2_) of activity loss during the exponential phase.

Inhibition of enzyme activity by L-Phe (0–50 mM) and L-homoArginine (0–50 mM) was measured in standard assay buffer, as previously described [51]. The IC_50_ was calculated from plots of residual enzyme activity against inhibitor concentration, using Origin plotting software (OriginLab). The nature of L-Phe inhibition was investigated using double-reciprocal plots of *v* versus substrate concentration, at various inhibitor concentrations. Integrated competition studies were carried out in standard assay buffer by premixing 0.1 mM pNPP with ATP (0–10 mM), PP_i_ (0–10 mM) or LPS (0–2 mg mL^−1^), before adding 200 µL of this mixture to 10 µL of enzyme preparation and measuring p-nitrophenol formation at pH 7.4.

### Zn^2+^ removal and reconstitution

The rate of Zn^2+^ dissociation was studied by pre-diluting the enzyme stock 10–10,000-fold in 50 mM Tris-HCl buffer, pH 7.4, containing 100 mM NaCl, that had been pretreated with BT Chelex 100 (Biolabs), which is a resin that efficiently chelates divalent ions. To avoid rebinding of dissociated Zn^2+^, diluted enzyme was incubated with 250 µM EDTA for increasing time intervals (0–360 min), during which time samples were removed and AP activity was measured in Chelex-treated 0.1 mM pNPP in 50 mM Tris-HCl buffer, pH 7.4, 100 mM NaCl. To avoid competitive inactivation by EDTA [29], samples were diluted 20-fold, to reduce EDTA concentration to 12.5 µM. Alternatively enzyme solutions were incubated at room temperature with Chelex to 30% of the total volume. After the indicated times, demetalated enzymes were incubated with 50 mM Tris buffer pH 7.4, containing 100 mM NaCl, supplemented with increasing concentrations of Zn^2+^ (0–20 µM), and the activity was measured in 0.1 mM pNPP in the Zn^2+^-reconstituted buffer [52].

### Statistical analysis

All quantitative measurements were carried out 3–5 times and calculated numbers are represented as the mean +/− SD. Likewise, figures were constructed using mean values ± SD, unless indicated differently, in which case single tracings are shown, representative of at least 3 experiments. Differences in inhibition by uncompetitive inhibitors and GndCl were statistically validated via an unpaired t-test and calculation of a two-tailed P-value. Differences in the inactivation rate of PLAP, IAP and ChimAP by GndCl or EDTA were statistically evaluated from the slope of linearized exponential plots of residual enzyme activity vs time, using GraphPad Prism.
